# Study of the Mechanical Properties and Microstructure of Spiral Tubes and Actuators for Controlled Extension Fabricated with Beryllium Bronze Strips

**DOI:** 10.3390/ma16206719

**Published:** 2023-10-17

**Authors:** Ruilong Lu, Jingtao Han, Zhanhua Li, Congfa Zhang, Jiawei Liu, Cheng Liu, Zhenqian Lang, Xiaoyan Ma

**Affiliations:** 1 School of Materials Science and Engineering, University of Science and Technology Beijing, Beijing 100083, China; 2 Guangzhou Sino Precision Steel Tube Industry Research Institute Co., Ltd., Guangzhou 511300, China; 3 School of Mechanical Engineering, Shijiazhuang Tiedao University, Shijiazhuang 050043, China; 4 Institute of Spacecraft System Engineering, China Academy of Space Technology, Beijing 100094, China

**Keywords:** STACER, beryllium bronze (QBe2.0), deployable boom, mechanical properties, microstructure

## Abstract

QBe2.0 strips were used to fabricate spiral tubes and actuators for controlled extension (STACERs) through the winding and stabilization method, which is a novel technique for obtaining STACERs. The raw strips and the STACERs were investigated using tensile tests and SEM for the mechanical properties and fractography observation, employing specialized test equipment for service performance, and via XRD, EBSD, and TEM were used to test the residual stress and microstructure evolution. The tensile strength/elongation for raw strips was 485.8 MPa/60%, while for STACERs, tensile strength increased by 834.67 MPa to 646 MPa, and the elongation rate decreased by 12% to 19.3%. The fractography showed that the fracture mode was ductile. The service performance tests indicated that STACERs obtained under 320 °C had a higher driving force, good pointing accuracy, and high bending stiffness, while the residual stress of raw strips was τ_xy_ = −6 MPa; for STACERs obtained between 290 °C and 350 °C, τ_xy_ decreased from −5 MPa to −74 MPa, then increased from −74 MPa to 21 MPa, and the optimum fabricating parameter was 320 °C + 2 h. The EBSD results showed that LABs and HABs for raw strips and STACERs at 320 °C + 2 h accounted for 3–97% and 24.5–75.5%, the grain sizes were 7.07 μm and 3.67 μm, and the twin fraction decreased from 57.3% to 31.8%, respectively. The KAM and Schmid factor maps indicated that the STACER was prone to recovering and recrystallizing. Coupled with the EBSD results, the TEM results indicated that the strengthening mechanism for raw strips is twinning strengthening, while that for STACER is grain-refining strengthening with a precipitation of the γ″ phase. It is a meaningful novelty that the relationship between the macro properties and microstructure has been elucidated.

## 1. Introduction

**Figure 1:**
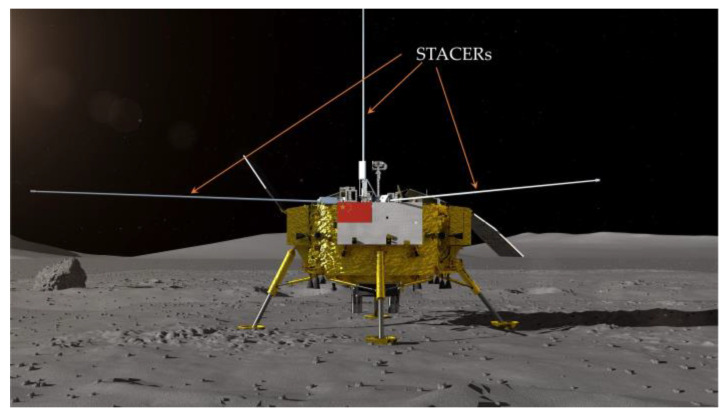
Schematic configuration of the lander for China’s Chang’E-4 Lunar’s exploration mission with deployable STACERs.

With the rapid development of spacecraft technology, increasing attention is being paid to the study and application of deployable mechanisms [1]. The focus on spiral tubes and actuators for controlled extension and retraction (STACERs), as typical one-dimensional linear deployable booms [2,3,4], is fueled by the heightened interest in aviation and space of researchers all around the world [5,6], such as in the case of the STACERs applied on the lander of China’s Chang’E-4 Lunar’s exploration mission [7], as shown in Figure 1.

**Figure 2:**
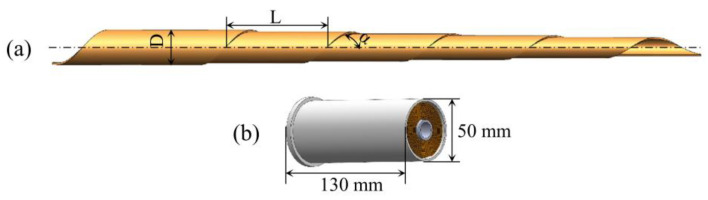
Schematic diagram of a STACER showing the (**a**) deploying state and (**b**) stowed state.

A STACER is a flat spring with a nearly constant diameter (D), fixed helical pitch (L), and fixed helix angle (α). It is usually obtained by rolling thin strips into a thin-wall tube-like boom with a sufficient stiffness and bending strength, as illustrated in Figure 2. To determine their optimum properties for specific applications, STACERs range in length from less than 1 m to more than 10 m, and range in diameter from 4 mm to 55 mm at the tip [8]. When stowed, a STACER is a very compact package; it is a coiled cylinder of thin strips screwed into the storage canister with the size of Φ 50 mm × 130 mm (Figure 2b). At deployment, it forms a spiral configuration with the initial coil winding out of the storage canister onto a cylindrical tip piece and grabbing the tip piece tightly; subsequently, the following coils stack up on the prior coil with a significant constant overlap under the fixed helical pitch [9,10], such that any section taken from the STACER would yield at least two layers of alloy strips, and the latter layer grips the former layer tightly with a force normal to the surface, resulting in significant inter-coil friction and preventing slipping between layers. A STACER can provide a deployment force from almost zero to more than 200 N due to the strain energy stored within itself without external force requirements.

Excellent mechanical properties and service performance are mostly dependent on the fabricating process and its specific parameters. The preparation methods publicly reported in the literature mainly comprise two types: (1) the compositing stretch and press bending (CSPB) method, proposed by Li et al. [11,12,13], and (2) the winding and stabilization method [14]. Li et al. elucidated the CSPB method in detail, including the forming principle, special-purpose equipment, preparing procedures, and obtained products with excellent properties. Kong et al. [15] conducted the analysis and verification of the deployed stiffness and illustrated the relationship between the parameters of the STACER and first natural frequency mode. Wu et al. [16] mainly focused on the flexible multibody dynamics model of STACERs and simulated their deployment; before the dynamics analysis and the deployment simulation, the experimental system was presented, although the forming method of the STACER was not given in detail (such as the winding specifics and the heat treatment parameters) and the used material was a stainless steel strip with a different thickness which is supposed to have poorer elastic properties than do strips of Co40NiCrMo alloy and CuBe2.0 alloy. Furthermore, the mandrel for rolling the strip on was tapered and not applicable for the fabrication of long components (the STACER in Wu’s study was less than 1 m long); this study further developed the aspects of the forming process, mechanical properties, and microstructure for the STACER of QBe2.0, since the Co40NiCrMo STACER was previously studied in detail [14].

Beryllium copper (QBe2.0), a typical precipitation-hardened alloy, can obtain a high strength close to that of steel after being subjected to an appropriate heat treatment process [17], and is widely used in a variety of fields. Decomposition and precipitation are proven to contribute to the strengthening and hardening [18] of a supersaturated solid solution of the beryllium copper matrix [19,20]. Many investigations have been conducted, aiming to clarify the phenomenon of phase transformation and precipitation sequences during the heat treatment process for Cu–Be alloys [21,22,23]. All of the literature mentioned in this section has illustrated the mechanisms of decomposition or precipitation [24,25,26] for Cu–Be alloys using technologies such as tensile experiments, XRD, and TEM, which mainly elaborate on the properties of specific materials themselves. Furthermore, for the adopted alloy strips, such as QBe2.0, as an excellent beryllium-containing alloy, the structural parts will be subject to irradiation by the ions or rays from outer space; hence, the study of microstructure evolution for this kind of alloy when irradiated bears profound significance. We aimed to carry out more exploration based on inspiration from some excellent prior research [27,28,29]. Building on the basis of the QBe2.0 material alloy, we conducted this study from another point of view by exploring the macro mechanical properties and the microstructure evolution of our target product—a STACER fabricated out of QBe2.0—through preparation procedures with different processing parameters.

The research aimed to explore a novel method at optimum parameters to obtain STACERs and to investigate the materials’ mechanical properties and service performance for the STACER components, in addition to determining the strengthening mechanism for the raw strips and the STACER at the microlevel. In this study, we reasonably designed the fabricating procedures for a STACER composed of QBe2.0, systematically tested mechanical characteristics such as tensile properties and residual stress distribution, and assessed its basic in-orbit service performances. The microstructure for STACERs of this type was observed using SEM, EBSD, and TEM characterization methods, and the strengthening mechanism was clarified.

## 2. Materials and Experimental Methods

### 2.1. Material

The material used for obtaining the STACER in this study was the beryllium bronze industrial brand type QBe2.0; the composition is detailed in Table 1. We utilized beryllium bronze strips with a thickness of 0.1 mm and a width of 127 mm.

### 2.2. Forming Procedures

**Figure 3:**
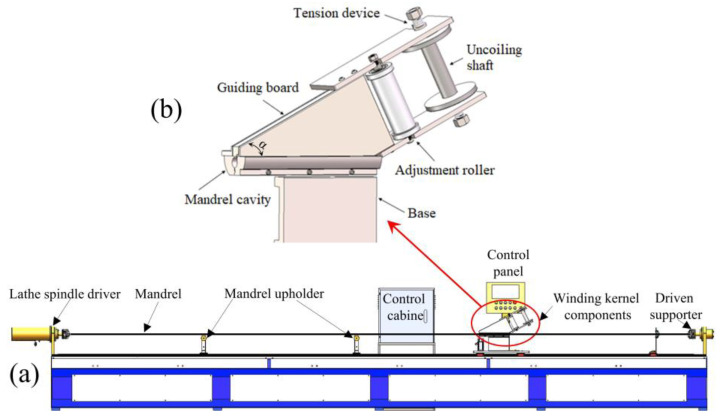
Schematic plot of specific equipment for the winding procedure. (**a**) Overall layout; (**b**) winding kernel component.

**Figure 4:**
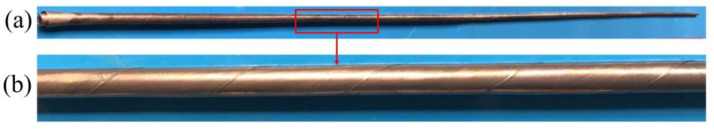
Physical appearance of a STACER obtained through the winding and stabilization process. (**a**) Overall appearance; (**b**) local appearance.

The winding and stabilization forming process was developed with consideration of the appearance configuration of the STACER component, the features of deploying action, and the necessary properties in practical applications for STACERs. The whole process can be divided into two continuous procedures: the winding procedure and stabilization procedure (vacuum heat treatment). The schematic plot of the specific equipment used for the winding procedure is shown in Figure 3. The mandrel was first gripped tightly in the three jaw chucks arranged at both ends of the mandrel cavity of the equipment; then, the kernel components were moved to the left side of the operation platform. The beryllium bronze strips were drawn out from the uncoiling shaft through the clearance of the guiding board and fixed on the mandrel, with the planar movement of the winding kernel components being set to a specific speed from left to right following the rotation of the lathe spindle driver, resulting in the beryllium bronze strips being wrapped around the mandrel with a constant helix angle (α), forming the preformed STACER. Preformed STACERs with different helix angles or diameters can be obtained by adjusting the feeding angle (α, as shown in Figure 3b) or alternating mandrels with different diameters. The vacuum heat treatment process was adopted for the stabilization procedure, in which the degree of the vacuum reached approximately 10^−5^ Pa to protect the components from oxidation. After the stabilization procedures, the beryllium bronze strips were gradually peeled from the mandrel, forming the final STACER components. A physical view of the STACERs is shown in Figure 4.

### 2.3. Tensile Tests and Fractography Observations

**Figure 5:**
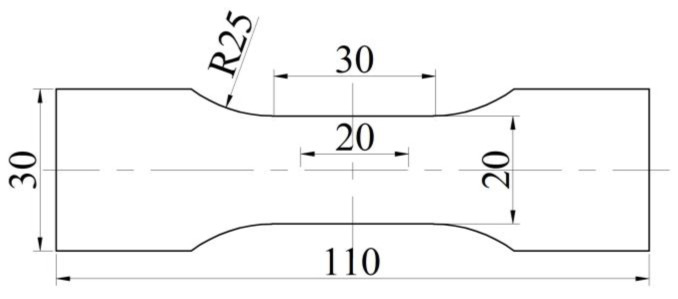
The dimensions of the specimen for room-temperature tensile tests (unit: mm).

Room-temperature tensile tests of original strips and STACERs prepared using different forming parameters were carried out by using a universal testing machine, the CMT-4204 (UTC 2017-042) universal testing machine, with the following specific forming parameters: a feeding angle for the winding process being fixed at 63 °, and the heat treatment parameters for stabilization procedure being 290 °C, 320 °C, and 350 °C, with holding for 1.5 h, 2 h, and 2.5 h, respectively. Room-temperature tensile specimens were cut from the original strips and the STACERs were obtained through the above stabilization parameters with the dimensions shown in Figure 5. The ZEISS Gemini SEM 500 apparatus was used to observe the fresh fracture surface morphology after the tensile tests, during which the working distance (WD) was set as 11.5 mm, and the extra-high tension (EHT) was chosen as 20.00 kV.

### 2.4. Residual Stress and Service Performance

**Figure 6:**
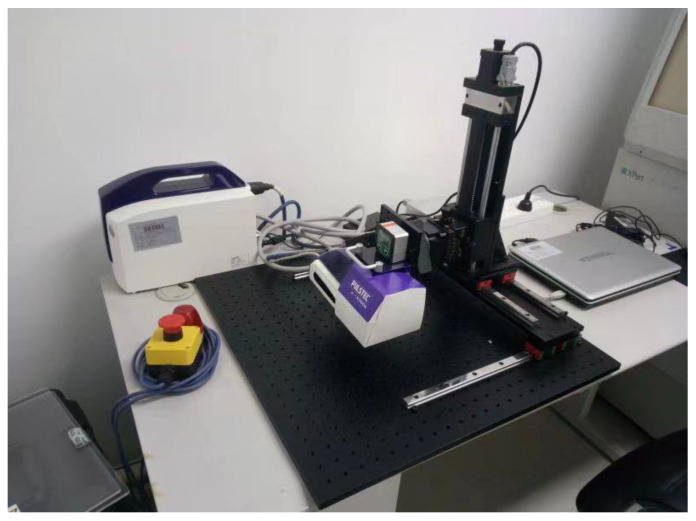
Pulstec μ-X360s X-ray residual stress analyzer.

In this study, residual stress measurements were conducted with a Pulstec μ-X360s X-ray residual stress analyzer from the Pulstec Industrial Co., Ltd. Hamamatsu, Japan (see Figure 6) based on the testing principle of the cosα method. The testing parameters were as follows: X-ray wavelength λ = 2.291 Å(10^−10^ m), an operating voltage of 30 kV, and an operating current of 2 mA. The Pulstec μ-X360s X-ray residual stress analyzer is a compact portable system with a two-dimensional area detector. The cosα method utilizes the whole Debye–Scherrer ring recorded on a two-dimensional detector taken using a single exposure of X-rays, in which the normal and shear stresses are determined simultaneously. The accuracy of the residual stress measurement of the cosα method has been confirmed to be equivalent to that of the sin^2^*Ψ* method for various metals; moreover, the simple optical system in the cosα method makes the analyzer smaller, lighter, and more convenient to use in on-site or field measurements [30]. In the testing process, the Kα doublet from the {220} plane’s family was used due to its high Bragg’s angle (2θ = 128.902°), providing a better accuracy of measurement; the surface of the STACERs was cleaned using ethanol with no extra treatment.

To meet the on-orbit performances, the STACERs were designed to be subject to service performance tests. A space environment mainly demands deploying stability, directivity characteristics, and anti-interference ability, which reflects the specific properties of the deploying self-driving force, pointing accuracy, and bending stiffness. The deploying self-driving force was tested during the deploying process (during which the STACER was not subject to any friction along its axis direction) at a certain deploying speed (1 m/min) equal to the deploying action on orbit, which is a key parameter guaranteeing the effective deployment of a STACER. Pointing accuracy and bending stiffness are supposed to indicate the straightness of the deploying STACERs to explore space signals in a given direction. During the pointing accuracy tests, the STACER was set perpendicular to the testing platform, deploying the STACER at the same speed as in the driving force tests, an the deviation angle from the tip to the bottom of the STACER was used to evaluate the pointing accuracy. For bending stiffness, the STACER was put on a testing platform with the axis direction parallel to the platform surface, moving the STACER out over the platform edge, and gently releasing the tip of the STACER. The value of bending stiffness was calculated using a method similar to that for the cantilever.

### 2.5. EBSD and TEM Analysis

The EBSD technique was conducted to illustrate the microstructures and the preferred orientations for the original strips and the STACER components. The testing specimens were obtained from the raw strips; STACERs were 5 mm × 5 mm in size, and the same as the original strips in thickness. The specimens were mechanically polished after grinding with 2000-grit sandpaper, and then electrolytically polished with 5% alcohol perchloric acid. The EBSD experiment was carried out on the Oxford Nordlys Max 3 apparatus, and the scanning step size was set as 0.4 μm. The TEM approach was used to explore the evolution of the microstructure in more detail, such as second-phase particles and twins. The TEM specimens were cut with a spark erosion cutter into disks of 3 mm in diameter and 0.1 mm in thickness, and pre-thinned to a 30 μm thickness. The thinned region was obtained using a Gatan 691 ion-beam thinning device, and a Mo self-supporting grid was used before carrying out the TEM experiments on a TECNAI F-200 field-emission transmission electron microscope (TEM) with an acceleration voltage of 200 kV.

## 3. Results and Discussions

### 3.1. Tensile Experiment and Fractography Observations Analysis

**Figure 7:**
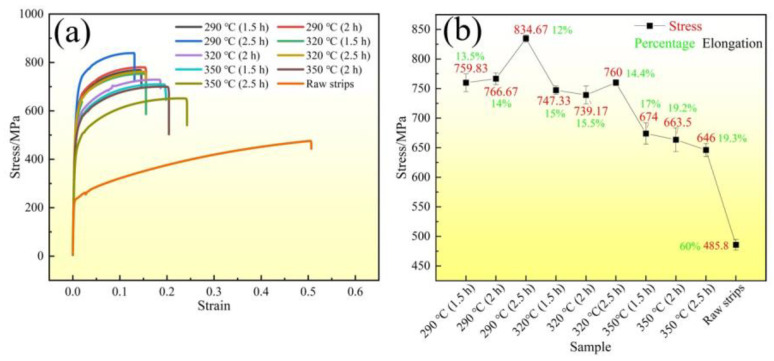
Stress–strain curves (**a**) and stress/elongation changes (**b**) for samples produced using different parameters.

The room-temperature tensile experiment results are shown in Figure 7. The specimens were raw QBe2.0 strips and STACERs fabricated using different stabilization (heat treatments) conditions (290 °C, 320 °C, and 350 °C, with holding for 1.5 h, 2 h, and 2.5 h, respectively). The raw strips were in a quenched state (soft state), which made them easy to be formed into different shapes, the tensile strength was 485.8 MPa, and the elongation was 60%, exhibiting sound formability. For the STACERs subject to different heat treatments, with the increase in temperature from 290 °C to 350 °C, the tensile strength decreased gradually from 834.67 MPa to 646 MPa, and the elongation rate increased from 12% to 19.3%, as shown in Figure 7b. The QBe2.0 alloy was typical precipitation-hardened alloy; therefore, second-phase particle decomposition and precipitation occurred during the heat treatment process, the strips strengthened to a certain degree between approximately 160 MPa and 350 MPa, and the rising heat temperature coupled with the prolonging of the holding time weakened the strength and promoted plasticity [31], which implied that the precipitation process may have been finished, and the recovery and recrystallization phenomenon proceeded.

**Figure 8:**
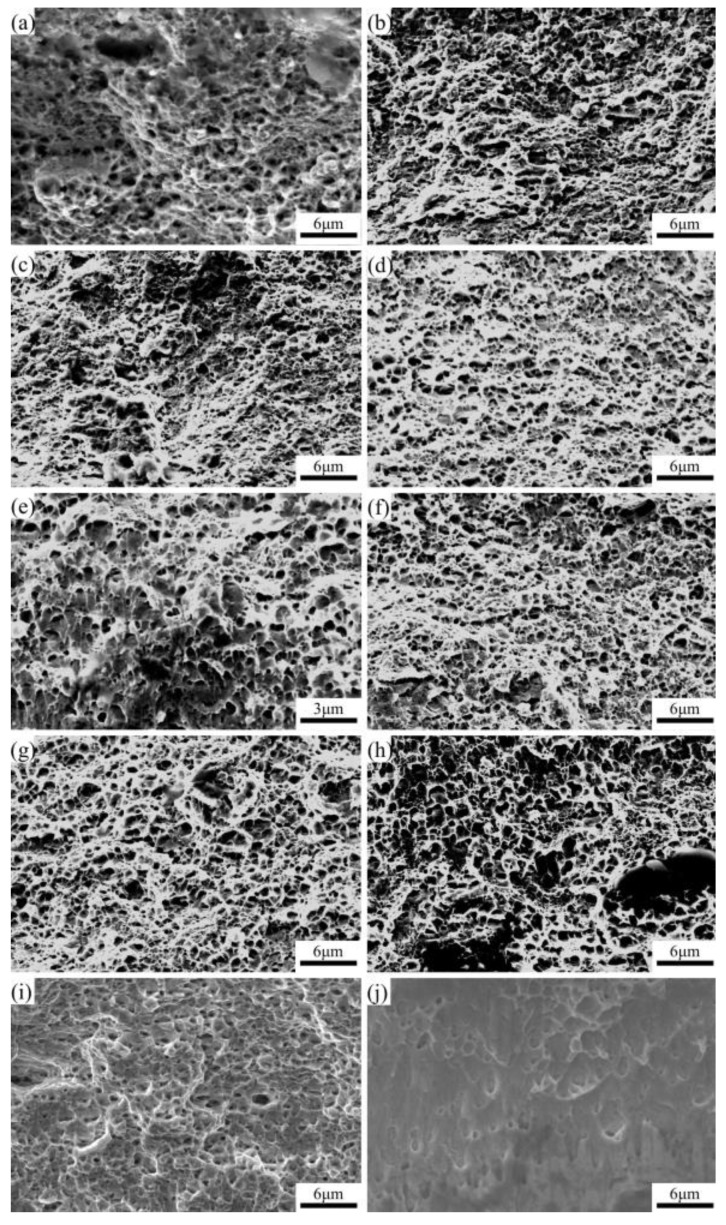
Tensile fracture morphology of STACERs obtained under different stabilization conditions: (**a**) 290 °C + 1.5 h; (**b**) 290 °C + 2 h; (**c**) 290 °C + 2.5 h; (**d**) 320 °C + 1.5 h; (**e**) 320 °C + 2 h; (**f**) 320 °C + 2.5 h; (**g**) 350 °C + 1.5 h; (**h**) 350 °C + 2 h; (**i**) 350 °C + 2.5 h, and (**j**) raw strips.

The fracture surface after the tensile experiment was observed using a SEM instrument. Figure 8a shows that the STACERs produced under the 290 °C + 1.5 h scheme exhibited a fracture surface that mainly consisted of a few cleavage facets with different sizes and continuous dimples. In Figure 8b,c, the microscopic fractography shown indicates that homogeneous dimples account for the majority of the scanning area, and the size and the depth of the dimples are associated with the number of void nucleation sites for cracks and the plasticity of the materials [32]. The appearance of facets in Figure 8a indicates that the ductility of the STACER under 290 °C + 1.5 h is worse than that under 290 °C + 2 h and 290 °C + 2.5 h. For the STACER stabilized under 320 °C, as shown in Figure 8d–f, the fracture morphologies consisted of relatively uniform dimples without facets, while the dimples were almost 1 μm in size and tended to merge on the surface of the dimples, showing locally flat and smooth features [33]; this implies that the plasticity of the STACER became better compared with that of the STACER under 290 °C. With the increase in the stabilization temperature from 320 °C to 350 °C, as shown in Figure 8g–i, the dimples merged much more and the fracture mode changed from the aggregation of the microvoids to slipping fracture or pure shearing [34]. Figure 8j shows the fractography of raw strips (quenched state); a lot of flat shallow dimples and spherical or bypassed particles were apparent in the matrix, which is typical ductile fracture, indicating that the raw strips had the best ductility among these samples. Briefly, the fracture morphologies accurately explain the law of tensile experiment curves at the micro-level. Coupled with curves from the tensile experiment, the STACERs showed smaller dimples, implying that the grain size was smaller than that of the raw strips; with the increase in the heating temperature and the prolonging of the holding time, the dimples were prone to expanding and tended to merge, which also implied the occurrence of recovery and recrystallization.

### 3.2. Service Performances and Residual Stress Analysis

The STACER is a rolled flat spring with a constant helical pitch and fixed diameter; thus, at deployment, the formation of the STACER started with initial coil winding out of the storage canister onto a cylindrical tip piece, which was slightly larger than the free coil diameter at the top of the STACER. To guarantee smooth deployment and precision during the service performance of the STACER, essential properties needed to be tested and confirmed. STACERs behave as a thin-walled tubes for minor displacement, with similar bending strengths and stiffnesses; if there is a large displacement, the coils slip, dissipating the strain energy, and the STACER serves as a friction damper. Furthermore, if the displacement ascends the limit, bucking phenomena occur, similarly to the case of any tube when subjected to strength beyond its yielding limit [35]. The main service performance aspects are as follows: driving force, pointing accuracy, and bending stiffness.

**Figure 9:**
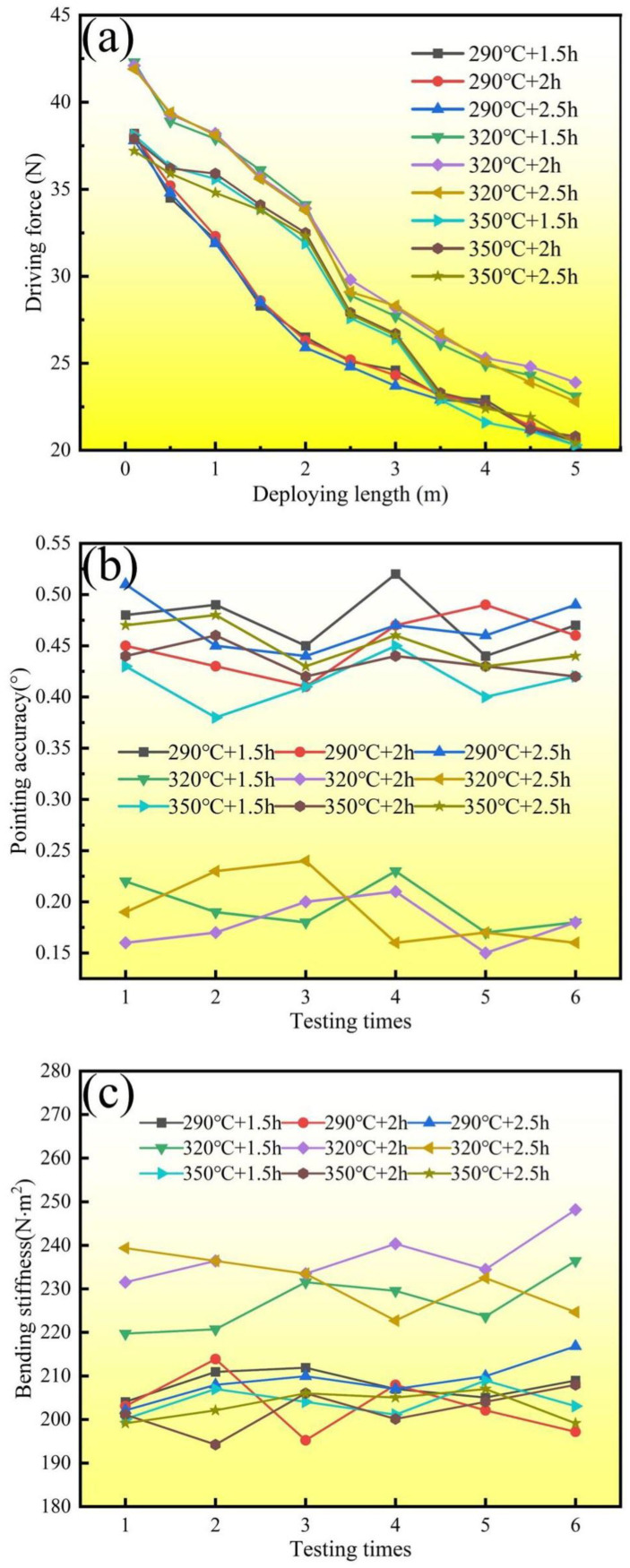
Service performance of STACERs obtained with different stabilization parameters: (**a**) driving force; (**b**) pointing accuracy; and (**c**) bending stiffness.

The driving force is a vital property for ensuring a sound STACER deployment process; then, the subsequent action and exploration process can be achieved. Stored strain energy serves as the effort needed to move the boom along its given path. The results of the driving force measurement are given in Table 2; the line and symbol graph is shown in Figure 9a. The table and the graph indicate that the driving force is more than 20 N, and with the increase in deploying length, the driving force decreases gradually from about 40 N to approximately 20 N. On the other hand, the STACERs stabilized under temperatures of 290 °C and 350 °C appeared to have a lower initial driving force and a lower driving force at a deploying length of 5 m compared with those of the STACERs stabilized at 320 °C, which implies that the STACERs stabilized under 320 °C showed relatively better performance when deploying.

The second service performance aspect that needed to be tested was pointing accuracy, which is essential to keeping the directivity exactly along the designed direction. Pointing accuracy is defined as a feature of the deviation angle of the tip piece being far away from the body of the spacecraft from the root part of the STACER fixed on the body of the spacecraft. As shown in Table 3 and Figure 9b, the deviation angles for STACERs stabilized under 290 °C were all around 0.45°, similar to the case of the STACERs stabilized under 350 °C. For the STACERs stabilized under 320 °C, the deviation angles were around 0.19°, for almost half of the above two types of STACERs; this indicates that the STACERs stabilized under 320 °C had a smaller degree of deviation, i.e., better directivity.

As a one-dimensional linear space-deployable mechanism, the deployed STACER can be defined as a cantilever beam, which will represent deflections due to its bending moment when a certain uniform load is imposed on it. Here, the imposed load was gravity. Although gravity has a minor in the space environment, the characteristics of bending stiffness also play a considerable role in the stability and the directivity of the STACER. Based on the theory of mechanics of materials [36], we conducted bending stiffness measurements for STACERs on our self-developed equipment. The maximum deflection value was determined and then substituted into Formula 1, where *q* is the distributed load (i.e., gravity, and the acceleration gravity, *g* = 9.81 m/s^2^). After calculating the *EI* (bending stiffness) through Formula 1, the data for bending stiffness were laid out in Table 4 and plotted in the scatter-mark graph depicted in Figure 9c. The results show that almost all bending stiffness values were over 200 N·m^2^; however, the STACERs stabilized under 320 °C had a larger bending stiffness around 230 N·m^2^ than did those under 290 °C and 350 °C, indicating that the STACERs stabilized under 320 °C demonstrated better performance in deploying stability and sound directivity.

(1) $$EI=\frac{qL^{4}}{8\delta_{B}}$$

In Equation (1), *δ_B_* is the maximum deflection of the beam, *q* is the distributed load (force per unit distance), *L* is the length of the STACER (cantilever beam), *E* is the elasticity, *I* is the inertia, and *EI* is the stiffness.

**Figure 10:**
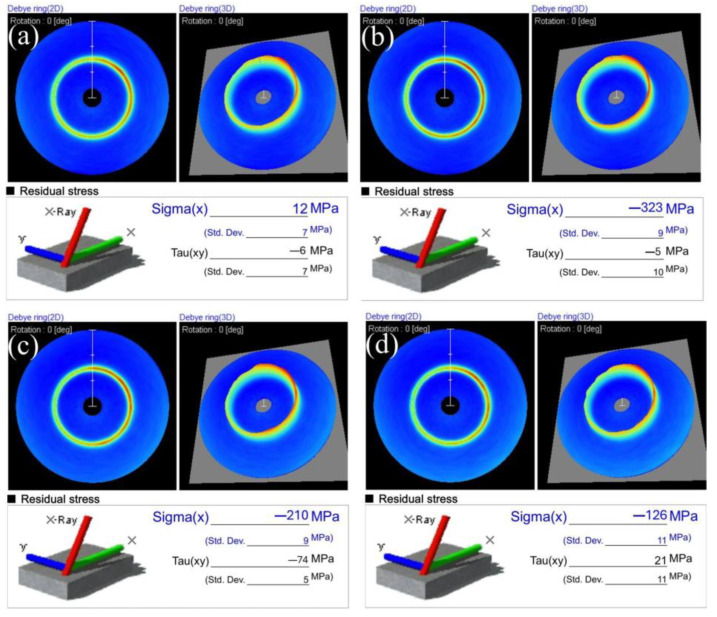
The Debye–Scherrer rings and residual stress for the raw strip and the STACERs stabilized under different heat treatment conditns: (**a**) raw strip; (**b**) 290 °C + 2 h; (**c**) 320 °C + 2 h; and (**d**) 350 °C + 2 h.

Based on the above tests for driving force, bending accuracy, and bending stiffness, holding times of 1.5 h, 2 h, and 2.5 h had no significant effect on those three kinds of service performances; the heating temperature had the most considerable effect. Thus, to explore the relationship between heating temperature and residual stress, we chose the STACERs obtained under a holding time of 2 h for each heating temperature to conduct the residual stress measurements using a 2D detector, the Pulstec μ-X360s X-ray residual stress analyzer from the Pulstec Industrial Co., Ltd. Hamamatsu, Japan. The results of the residual stress measurement are depicted in Figure 10, including the Debye–Scherrer rings [37] and the values of residual stress (Sigma(x):σ_x_, Tau(xy):τ_xy_) for raw QBe2.0 strips (Figure 10a), and winding and stabilization STACERs stabilized under the condition of 290 °C + 2 h (Figure 10b), 320 °C + 2 h (Figure 10c), and 350 °C + 2 h (Figure 10b). Figure 10a shows that the residual stress components, σ_x_ = 12 MPa, and τ_xy_ = −6 MPa for the as-received raw strips were subjected to sheet leveling and stress relief; thus, the values of residual stress were located around zero. For the strip components fabricated into STACERs, the value of the residual stress component, σ_x_, increased from −323 MPa to −126 MPa with the increase in heating temperature from 290 °C to 350 °C, while the residual stress component, τ_xy_, decreased from −5 MPa to −74 MPa, and then increased from −74 MPa to 21 MPa with the increase in heating temperature from 290 °C to 350 °C. This indicated that the residual stress component, σ_x_, was compressive stress and was relieved to some extent with the increase in heating temperature. The residual stress component, τ_xy_, decreased to −74 MPa (larger absolute value), and was then relieved to the tensile stress. For this specific STACER configuration, τ_xy_ plays the vital role of keeping its shape, keeping its compact configuration, and catering to the service performance, because τ_xy_ as the compressive stress component guaranteed that the latter layer tightly gripped the former layer of the STACER part; the STACERs obtained under the condition of 320 °C + 2 h had a larger compressive stress, and coupled with the service performance analysis above, the residual stress measurement accurately illustrated the principle that the STACERs obtained under conditions of 320 °C + 2 h demonstrated better service performance.

The results of residual stress measurements indicated that the residual stress components, σ_x_, had been released to some degree after the stabilization process; for the winding procedure comprising cold forming, a lower heating temperature (290 °C) strengthened the materials, and the residual stress component, τ_xy_, did not reach the maximum, which is a key indicator of the tightness between the layers.

Upon the measurements and analysis of driving force, pointing accuracy, bending stiffness, and verification via the residual stress measurement, we could conclude that the STACERs stabilized under 320 °C and for 2 h exhibited superior performances; from the view of the study of the material, most of the macro properties should be elucidated from microstructure evolution [37,38,39,40,41]. For this reason, we conducted electron backscatter diffraction (EBSD) measurements and transmission electron microscopy (TEM) tests for the as-received raw strips and developed a STACER stabilized under 320 °C + 2 h for more explanation on the micro level.

### 3.3. Electron Backscatter Diffraction Analysis

**Figure 11:**
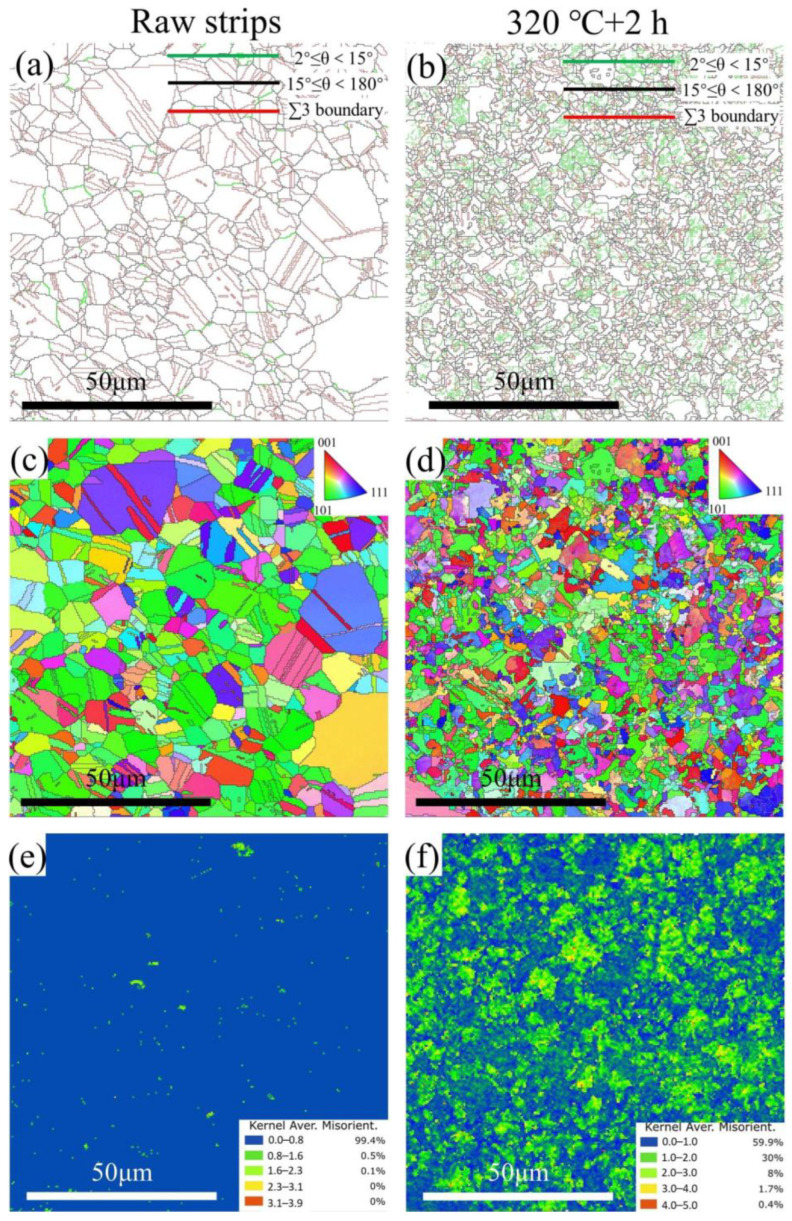

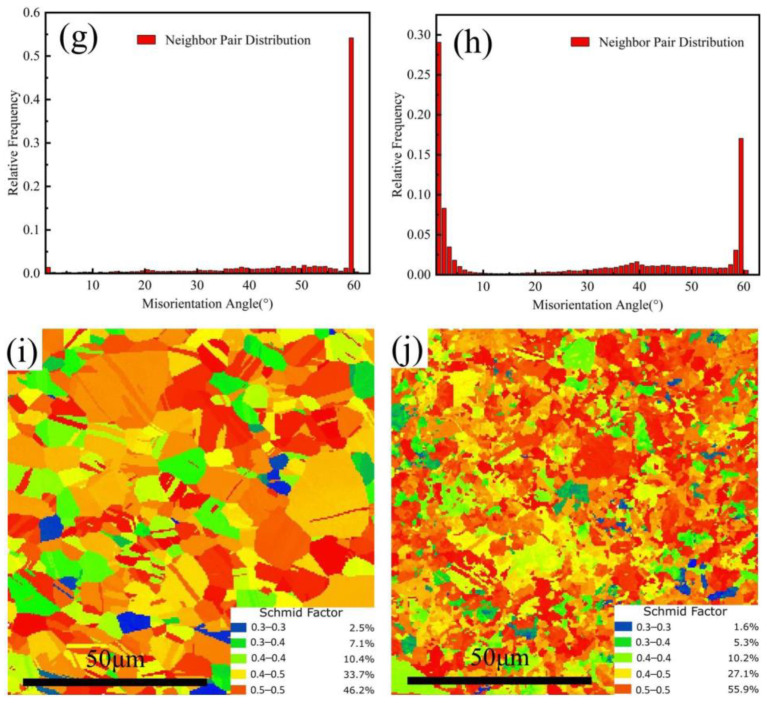
The results of the EBSD measurements: GB (**a**,**b**); IPF (**c**,**d**); KAM (**e**,**f**); MAD (**g**,**h**); and Schmid Factor (**i**,**j**) maps of raw strips (**a**–**i**) and winding and stabilization STACER (320 °C + 2 h) (**b**–**j**) specimens.

The results of the electron backscatter diffraction (EBSD) measurement are shown in Figure 11; the grain boundary (GB) maps, inverse pole figures (IPFs), kernel average misorientation (KAM) maps, and misorientation angle distribution (MAD) maps were constructed from the original data using the AZtecCrystal 2.1 software from Oxford Instruments (Abingdon, UK). Figure 11a,b shows the GBs and the size and shape of grains; the low-angle boundaries (LABs, 2° ≤ θ < 15°) and the high-angle boundaries (HABs, 15° ≤ θ < 180°) accounted for 3–97% and 24.5–75.5%, respectively; the special grain boundary (∑3(<111>60°), i.e., twin boundaries) accounted for 57.3% and 31.8% for raw strips and the STACER stabilized under 320 °C + 2 h, respectively. The average grain sizes, determined using the equivalent circle diameter, were 7.07 μm and 3.67 μm, respectively, as shown in Table 5. The GB maps and the grain size distribution indicated that recrystallization occurred in the microstructure of the STACER stabilized under 320 °C + 2 h. The twins decreased during the winding and stabilization process of obtaining the STACER, the strengthening mechanism transformed from twinning strengthening to grain refinement strengthening, and for the misorientation angle distribution (MAD) histograms (shown in Figure 11g,h), the twins, i.e., ∑3(<111>60°), dominated the specimen, while in the STACER specimen, the misorientation exhibited a two-peak phenomenon, implying that recovery and recrystallization occurred during the fabrication process of the STACER.

KAM maps are shown in Figure 11e,f, from which the values of KAM were approximately (99.4%) located in the range of 0~0.8 for the raw strips; as for the STACER, the KAM values located in the range of 0~1.0 accounted for 59.9%, and those in the range of 1~2 and 2~3 accounted for 30% and 8%, respectively. The values of KAM are usually adopted to judge orientation differences between adjacent scanning points during EBSD measurements, corresponding to the degree of residual plastic strain, resulting in the storage of plastic strain energy in the alloy, ensuring sound plastic properties for the STACER. The Schmid factor is a variable characterizing the ratio of shear stress, resolved on the plane and in the direction of the relevant slip system, to normal applied stress. Notably, the Schmid factor in the STACER stabilized under 320 °C + 2 h in the range of 0.5~0.5 (55.9%) was higher than that in the raw strips (46.2%), which implies that the slip system in the STACER was more easily activated [42,43,44], this being more conducive to the occurrence of recovery and recrystallization, and the grain size of the specimen of STACER stabilized under 320 °C + 2 h was exhibited as about half of that in the raw strips (3.67 μm to 7.07 μm). This further indicated that the precipitation proceeded adequately, forming many more nucleus sites than did the raw strips, resulting in the occurrence of recovery and recrystallization. The strengthening mechanism transformed from twinning strengthening in raw strips to the grain-refining strengthening mechanism in STACERs.

### 3.4. Transmission Electron Microscopy Analysis

**Figure 12:**
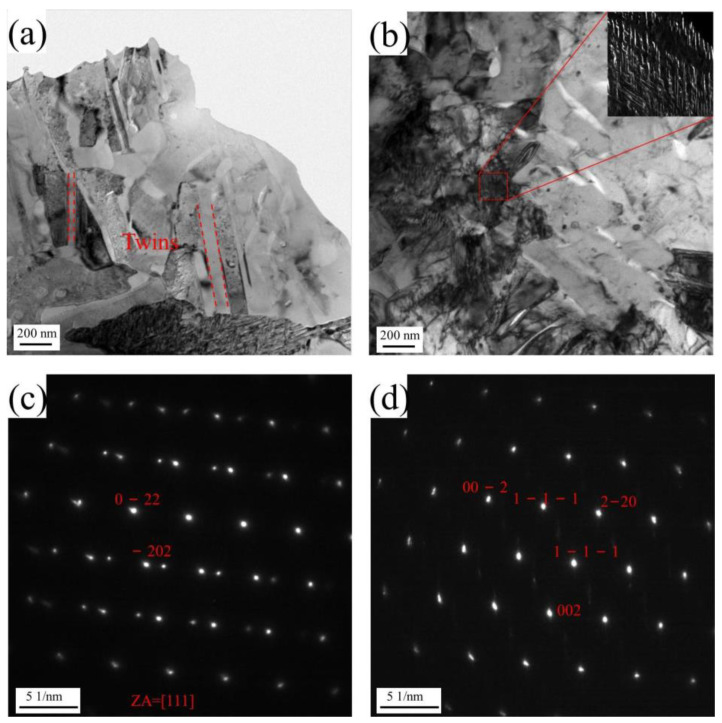
Results of the TEM measurements. Raw strips (**a**); STACER (320 °C + 2 h) (**b**); SAED for twins and diffraction spots of the substrate at the crystal axis of [111] (**c**); and SEAD for the streak-shaped patterns (**d**) of the QBe2.0 alloy strips.

Transmission electron microscopy (TEM) measurements were conducted on the raw strips and the STACER stabilized under 320 °C + 2 h; bright-field images and their selected area electron diffraction (SAED) images are depicted in Figure 12. As a type of basal FCC structure and age-hardening alloy, the solid solution becomes supersaturated after being held at high temperatures, and the excess vacancies and solute atoms tend to precipitate out in some form. The precipitation of the Cu–Be alloy consisted of the spinodal decomposition and transformation of a series of metastable phases. Figure 12a shows the larger twin and grain sizes in the map, which are consistent with the EBSD results, as shown in Figure 11a,c. For the crystal axis [111], the diffraction spots of the substrate were (0–22) and (−202), and the diffraction spots of twins were observed. When the raw strips were fabricated into a STACER (including the winding and stabilization process, i.e., bending and heat treatment procedures), the strips were subjected to plastic deformation and relatively low-temperature heat treatment, and the plastic strain energy stored in the alloy substrate was relieved to some degree; a striated microstructure of the alloy can be observed in the sub-figure of Figure 12b, indicating a diffuse basket weave of light and dark contrast, mainly present in the morphology of slip lines and slip stages. According to a previous study [26], the striation microstructure may also be attributed to solute segregation in the strained field. Some streak-shaped patterns along the (002)α direction are observable in the SAED of Figure 12d. According to previous research performed by Rioja and Laughlin [45], at the intensity maxima near the 2/3 (002)α and 1/2 (202)α reciprocal lattice positions, there are considered to be features of γ″ precipitates, which are usually believed to have a body-centered tetragonal (BCT) structure [17,46], containing a Be atom at the body center, with the lattice parameters a = b = 0.253 nm and c = 0.29 nm. From the testing results and analysis, we could verify the principle that the strengthening mechanism in the raw strips was twinning strengthening, while the strengthening mechanism in the specific STACER stabilized under 320 °C + 2 h was grain-refining strengthening, with some precipitation of second-phase particles, γ″.

## 4. Conclusions

The as-received raw strips of QBe2.0 and the STACERs obtained via the winding and stabilization method were investigated from the aspects of tensile properties, fractography, the service performances of the STACERs, residual stress, and microstructure (EBSD and TEM), aiming to elucidate the relationship between macro properties and microstructure for the STACER components obtained through this specific method. The main findings are described as follows:

(1) The tensile strength/elongation for raw strips was 485.8 MPa/60%, while for STACERs stabilized from 290 °C to 350 °C with holding for 1.5 h to 2.5 h, tensile strength decreased gradually from 834.67 MPa to 646 MPa, and the elongation rate increased from 12% to 19.3%. Fractography showed that the fracture mode was ductile.
(2) The STACER obtained under 320 °C demonstrated sound service performances. The residual stress measurement showed that the STACER obtained under 320 °C + 2 h had relatively higher absolute σ_x_ and τ_xy_ values; the τ_xy_ value, especially, is vital for the service performance of STACERs. The optimum parameter for fabricating STACERs was set as 320 °C + 2 h.
(3) The LABs and HABs for the raw strip and STACER of 320 °C + 2 h accounted for 3%-97% and 24.5–75.5%, the grain sizes were 7.07 μm and 3.67 μm, and the twin fraction decreased from 57.3% to 31.8%, respectively. The KAM and Schmid factor maps indicated that the substrate of the STACER of 320 °C + 2 h was prone to recovery and recrystallization. The TEM test confirmed that the strengthening mechanism for raw strips was twinning strengthening while for STACER, it was grain-refining strengthening, with some precipitation of second-phase particles, γ″.

## Figures and Tables

**Table 1:** Chemical compositions of beryllium bronze (QBe2.0) strips (wt.%).

Al	Be	Cu	Fe	Ni	Pb	Si
0.066	1.96	96.99	0.11	0.25	0.0008	0.13

**Table 2:** Data of driving force for STACERs under different forming parameters.

Specimen	Driving Force (N)										
	0.1 m	0.5 m	1 m	1.5 m	2 m	2.5 m	3 m	3.5 m	4 m	4.5 m	5 m
290 °C + 1.5 h	38.2	34.5	32.1	28.3	26.5	25.1	24.6	23.1	22.9	21.3	20.5
290 °C + 2 h	38.1	35.2	32.3	28.6	26.3	25.2	24.3	23.2	22.6	21.4	20.6
290 °C + 2.5 h	37.8	34.8	31.9	28.5	25.9	24.8	23.7	22.9	22.7	21.2	20.3
320 °C + 1.5 h	42.3	38.9	37.9	36.1	34.1	28.9	27.7	26.1	24.9	24.3	23.1
320 °C + 2 h	42.1	39.3	38.2	35.7	33.9	29.8	28.2	26.5	25.3	24.8	23.9
320 °C + 2.5 h	41.9	39.4	38.1	35.6	33.8	29.1	28.3	26.7	25.1	23.9	22.8
350 °C + 1.5 h	38.1	36.3	35.6	33.9	31.9	27.6	26.4	22.9	21.6	21.1	20.3
350 °C + 2 h	37.9	36.2	35.9	34.1	32.5	27.9	26.7	23.3	22.7	21.2	20.8
350 °C + 2.5 h	37.2	35.9	34.8	33.8	32.3	27.8	26.6	23.1	22.4	21.9	20.4

**Table 3:** Data of pointing accuracy for STACERs under different forming parameters.

Specimen	Pointing Accuracy (°)						
	1	2	3	4	5	6	Average
290 °C + 1.5 h	0.48	0.49	0.45	0.52	0.44	0.47	0.475
290 °C + 2 h	0.45	0.43	0.41	0.47	0.49	0.46	0.452
290 °C + 2.5 h	0.51	0.45	0.44	0.47	0.46	0.49	0.470
320 °C + 1.5 h	0.22	0.19	0.18	0.23	0.17	0.18	0.195
320 °C + 2 h	0.16	0.17	0.20	0.21	0.15	0.18	0.178
320 °C + 2.5 h	0.19	0.23	0.24	0.16	0.17	0.16	0.192
350 °C + 1.5 h	0.43	0.38	0.41	0.45	0.40	0.42	0.415
350 °C + 2 h	0.44	0.46	0.42	0.44	0.43	0.42	0.435
350 °C + 2.5 h	0.47	0.48	0.43	0.46	0.43	0.44	0.452

**Table 4:** Data of bending stiffness for STACERs obtained under different forming parameters.

Specimen	Bending Stiffness (N·m^2^)						
	1	2	3	4	5	6	Average
290 °C + 1.5 h	204.05	210.92	211.90	206.99	205.03	208.95	207.97
290 °C + 2 h	203.07	213.86	195.22	207.97	202.09	197.18	203.23
290 °C + 2.5 h	202.09	207.97	209.93	206.99	209.93	216.80	208.95
320 °C + 1.5 h	219.74	220.73	231.52	229.55	223.67	236.42	226.94
320 °C + 2 h	231.52	236.42	233.48	240.35	234.46	248.19	237.40
320 °C + 2.5 h	239.36	236.42	233.48	222.69	232.50	224.65	231.52
350 °C + 1.5 h	200.12	206.99	204.05	201.11	208.96	203.07	204.05
350 °C + 2 h	201.11	194.24	206.03	200.12	204.05	207.97	202.09
350 °C + 2.5 h	199.14	202.09	206.01	205.03	206.99	199.14	203.07

**Table 5:** Grain boundary fraction and the average size of grains for raw strips and winding and stabilization STACER (320 °C + 2 h).

Specimen	LABs (%)	HABs (%)	∑3(<111>60°) (%)	Average Grains Size (μm)
Raw strips	3	97	57.3	7.07
320 °C + 2 h	24.5	75.5	31.8	3.67

## Data Availability

Data will be made available on request.
